# Ultrasound-guided hydrostatic reduction of intussusception: comparison of success rates between subspecialized pediatric radiologists and non-pediatric radiologists or radiology residents

**DOI:** 10.1007/s00431-023-04987-1

**Published:** 2023-05-06

**Authors:** Luka Pušnik, Peter Slak, Stevan Nikšić, Abbey J. Winant, Edward Y. Lee, Domen Plut

**Affiliations:** 1 Institute of Anatomy, Faculty of Medicine, University of Ljubljana, Ljubljana, Slovenia; 2 Clinical Radiology Institute, University Medical Center Ljubljana, Ljubljana, Slovenia; 3 Department of Radiology, Faculty of Medicine, University of Ljubljana, Ljubljana, Slovenia; 4 Department of Radiology, Boston Children’s Hospital, Harvard Medical School, Boston, MA USA

**Keywords:** Ileocolic intussusception, Invagination, Enema, Ultrasonography, Pediatric radiology

## Abstract

Ileocolic intussusception is the most common cause of intestinal obstruction in children under two years of age. Treatment in most cases is radiologically guided reduction. In Slovenia, ultrasound (US)-guided hydrostatic reduction is currently the standard of care. The purpose of this study was to compare the success rate of US-guided hydrostatic reduction when performed by subspecialty-trained pediatric radiologists, non-pediatric radiologists, or radiology residents. We retrospectively analyzed medical records of patients with ileocolic intussusception who underwent US-guided hydrostatic intussusception reduction at University Medical Centre Ljubljana between January 2012 and December 2022 (*n* = 101). During regular daily working hours, the reduction was performed by pediatric radiologists. After hours (evenings and overnight), pediatric radiologists, non-pediatric radiologists, or radiology residents performed the reduction procedure. Patients were divided into three groups based on the operator performing the procedure. Data was analyzed using the chi-square test. Pediatric radiologists had thirty-seven (75.5%) successful first attempts, non-pediatric radiologists had nineteen (76.0%), and radiology residents had twenty (74.1%). There was no statistically significant difference in the success rate of ileocolic intussusception reduction depending on the operator who performed the procedure (*p* = 0.98). No perforation was observed in either group during the reduction attempts.

*  Conclusion*: Our results demonstrate that US-guided hydrostatic reduction is a reliable and safe procedure that achieves good results even in the hands of less experienced, however appropriately trained, radiologists. The results should encourage more medical centers to consider the implementation of US-guided hydrostatic reduction of ileocolic intussusception.

**Table:** 

**What is Known:** *• US-guided hydrostatic reduction is a well-established method of treatment for ileocolic intussusception in children.* *• The results regarding the influence of operator’s experience with the procedure on its success rate are scarce and contradictory.*	
**What is New:** *• US-guided hydrostatic intussusception reduction is a reliable and safe technique that achieves similar success rates when performed by experienced subspecialized pediatric radiologists or less experienced but trained operators such as non-pediatric radiologists and radiology residents.* *• The implementation of US-guided hydrostatic reduction in general hospitals without subspecialized pediatric radiologists could improve patient care by increasing access to radiologically guided reduction and simultaneously decreasing the time to reduction attempts.*

**What is Known:**

*• US-guided hydrostatic reduction is a well-established method of treatment for ileocolic intussusception in children.*

*• The results regarding the influence of operator’s experience with the procedure on its success rate are scarce and contradictory.*

**What is New:**

*• US-guided hydrostatic intussusception reduction is a reliable and safe technique that achieves similar success rates when performed by experienced subspecialized pediatric radiologists or less experienced but trained operators such as non-pediatric radiologists and radiology residents.*

*• The implementation of US-guided hydrostatic reduction in general hospitals without subspecialized pediatric radiologists could improve patient care by increasing access to radiologically guided reduction and simultaneously decreasing the time to reduction attempts.*

## Introduction

Ileocolic intussusception is the most common cause of intestinal obstruction in infants and small children under two years of age. The condition develops when the terminal ileum telescopes through the ileocecal valve into the colon [1]. In addition to mechanical bowel obstruction, this invagination of the small intestine into the large bowel often compromises the vascular supply of the bowel, resulting in ischemia that, if prolonged, can lead to necrosis and rarely, intestinal perforation, peritonitis, shock, and even death [2]. Early diagnosis and timely treatment of intussusception are imperative for preserving bowel integrity and preventing complications and mortality. In most cases, the treatment of ileocolic intussusception is hydrostatic or pneumatic reduction under imaging guidance [3].

Prompt reduction of ileocolic intussusception after diagnosis is therefore crucial for preventing complications [4]. At our institution, hydrostatic enema under ultrasound (US) guidance is performed to reduce ileocolic intussusception and all radiologists (including subspecialized pediatric radiologists and non-pediatric radiologists) and radiology residents who participate in off-regular-hour coverage are trained to perform this procedure. During regular working hours, the subspecialized pediatric radiologists perform this procedure, while during the off-regular-hour coverage, pediatric radiologists, non-pediatric radiologists, or radiology residents perform this procedure.

To date, a paucity of data exists regarding the complexity of performing the different reduction procedures and the influence of the operator’s experience on the success rate of the procedure [5–8]. In order to elucidate this limitedly researched topic, we performed a retrospective study with the aim to compare the success rate of US-guided hydrostatic intussusception reduction between three independent groups: the subspecialized pediatric radiologists who perform this procedure during regular working hours and the non-pediatric radiologists or radiology residents who perform this procedure during 24/7 duty. We hypothesized that US-guided hydrostatic intussusception reduction is equally efficient and safe even in the hands of less experienced operators such as non-pediatric radiologists and radiology residents.

## Materials and methods

### Institutional review board approval

Our institutional review board approved this retrospective review of patients’ medical records. Informed consent was waived because of the retrospective nature of this study. Patient confidentiality was maintained in accordance with national standards to protect sensitive patient health information. The research was conducted following the Helsinki Declaration.

### Patients

In our study, we retrospectively evaluated all pediatric patients (age: 0–17 years) who were diagnosed with ileocolic intussusception and underwent US-guided hydrostatic intussusception reduction at the University Medical Centre Ljubljana between January 2012 and December 2022. The data collected included demographic data, the outcome of the intervention, the presence of a lead point, operator data, and the number of recurrence episodes. Patients were divided into three groups based on the operator performing the procedure. The first group included children in whom the ileocolic intussusception reduction attempt was performed by a subspecialty-trained pediatric radiologist. Accordingly, this group was comprised of patients who presented during regular working hours or during 24/7 duty hours if a pediatric radiologist was on duty. The second and third groups included patients in whom non-pediatric radiologists or radiology residents, respectively, performed the intussusception reduction procedure. The second and third groups consisted entirely of children who presented during 24/7 duty hours when there was not a pediatric radiologist on duty.

### Qualification of the operators

The group of pediatric radiologists was comprised of board-certified radiologists who have been performing a full-time continuous clinical activity in the field of pediatric radiology for a minimum of 1 year. The group of non-pediatric radiologists comprised board-certified radiologists who perform their clinical activity in other fields of radiology (i.e., adult abdominal, adult cardiothoracic, or adult musculoskeletal radiology). The group of radiology residents comprised radiology residents who completed a considerable amount of training, including five months of ultrasound, two months of pediatric radiology, six months of abdominal radiology, and one month of emergency radiology training among others. During the US training, the residents gain in-depth knowledge of ultrasonography, daily performing around 15 abdominal US examinations under supervision. During the pediatric radiology training, the topic of diagnosing and reducing ileocolic intussusception is exhaustively taught in a form of a seminar. During this time, the resident also accompanies and observes the pediatric radiologist performing the procedure if such a situation occurs. Additionally, before being deemed qualified for the 24/7 duty service, the residents need to pass an institutional exam in emergency radiology.

### Diagnostic equipment

The US machines used for the US guidance of ileocolic intussusception reduction included a Mindray DC-70 US machine (Mindray Medical International Limited, Shenzhen, China) equipped with 12–3 MHz linear transducer (L12-3e) and 5.7–1.3 MHz convex transducer (C5-2e); Toshiba Aplio 500 (Canon Medical Systems Corporation, Otawara, Japan) equipped with 12–6.2 MHz linear transducer (PLT-805AT) and 6–1.9 MHz convex transducer (PVT-375BT); and Toshiba Xario (Canon Medical Systems Corporation, Otawara, Japan) equipped with 12–6.2 MHz linear transducer (PLT-805AT) and 6–1.9 MHz convex transducer (PVT-375BT).

### Procedure

**Fig. 1:**
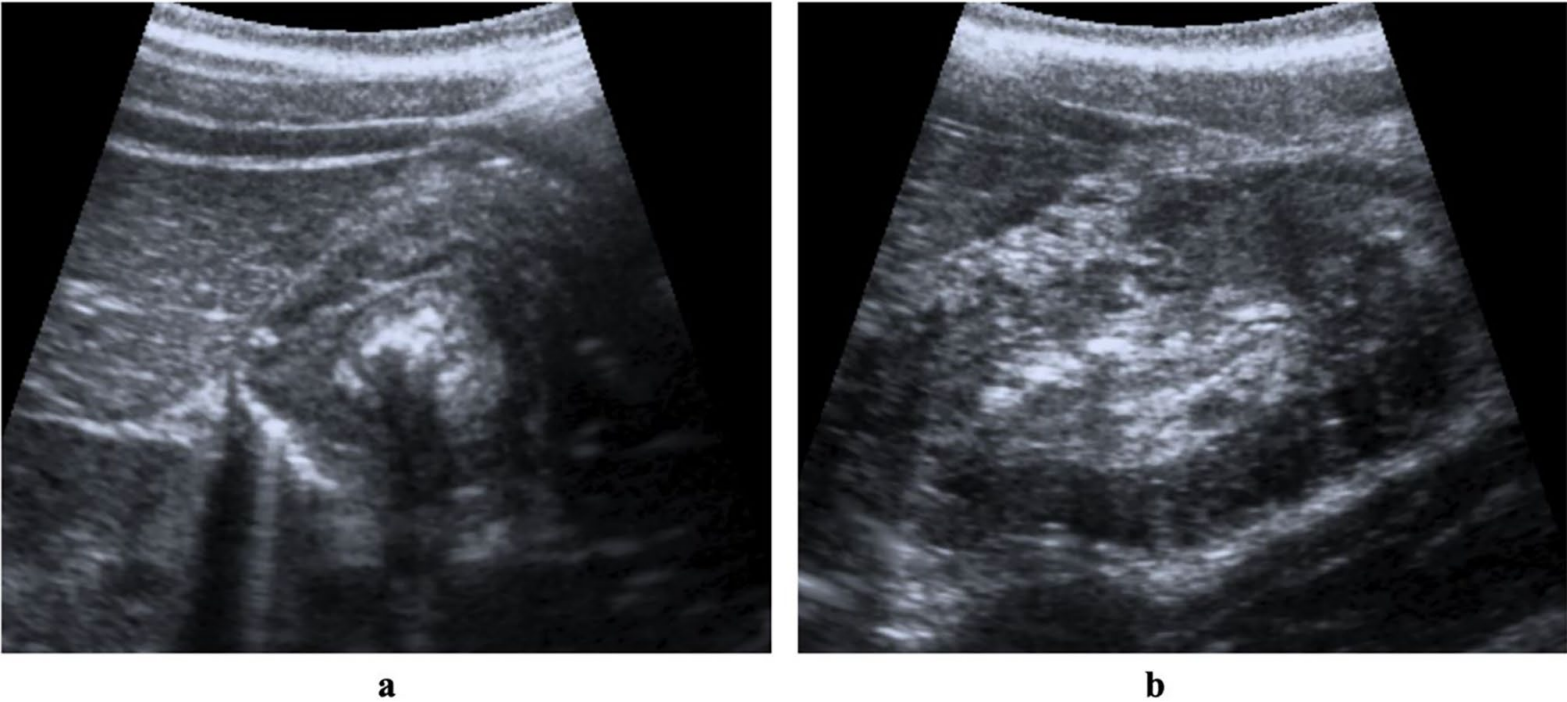
Right upper quadrant grayscale ultrasound images. **a** The typical bowel-within-the-bowel US appearance of ileocolic intussusception in transverse plane (doughnut or target sign). **b** The typical appearance in the longitudinal plane (pseudokidney sign)

After ileocolic intussusception was diagnosed (Fig. 1), the child had a surgical consultation to decide on the method of treatment. The first-line treatment for most cases of ileocolic intussusception is radiologic reduction; however, children who present with clinical signs of peritonitis, shock, or perforation are not candidates for radiologically guided reduction. After the decision was made to attempt a radiologic reduction, a team composed of a radiologist, a surgeon, and a nurse was assembled to perform the procedure.

The procedure is performed in an US room, usually in the same room as the initial US to diagnose ileocolic intussusception was performed. At our institution, the procedure has been traditionally performed without anesthesia or sedation. Although it may be contended that the process is distressing and uncomfortable for the child, there is ambiguity regarding the advantages of using sedation or general anesthesia during the reduction procedure, and its application differs significantly among different institutions [4]. A rectal tube (without the use or inflation of a balloon) is used to apply the saline into the child’s bowel. Warm saline is put in a cistern on a drip stand at a height of 1–1.5 m above the child and connected to the rectal tube. Before starting the procedure, the radiologist re-images the abdomen with the US to re-confirm the presence of ileocolic intussusception and to identify its distal ending in the colon. At this point, the rectal tube is inserted into the child’s rectum by a surgeon and the inflow of warm saline is started.

**Fig. 2:**
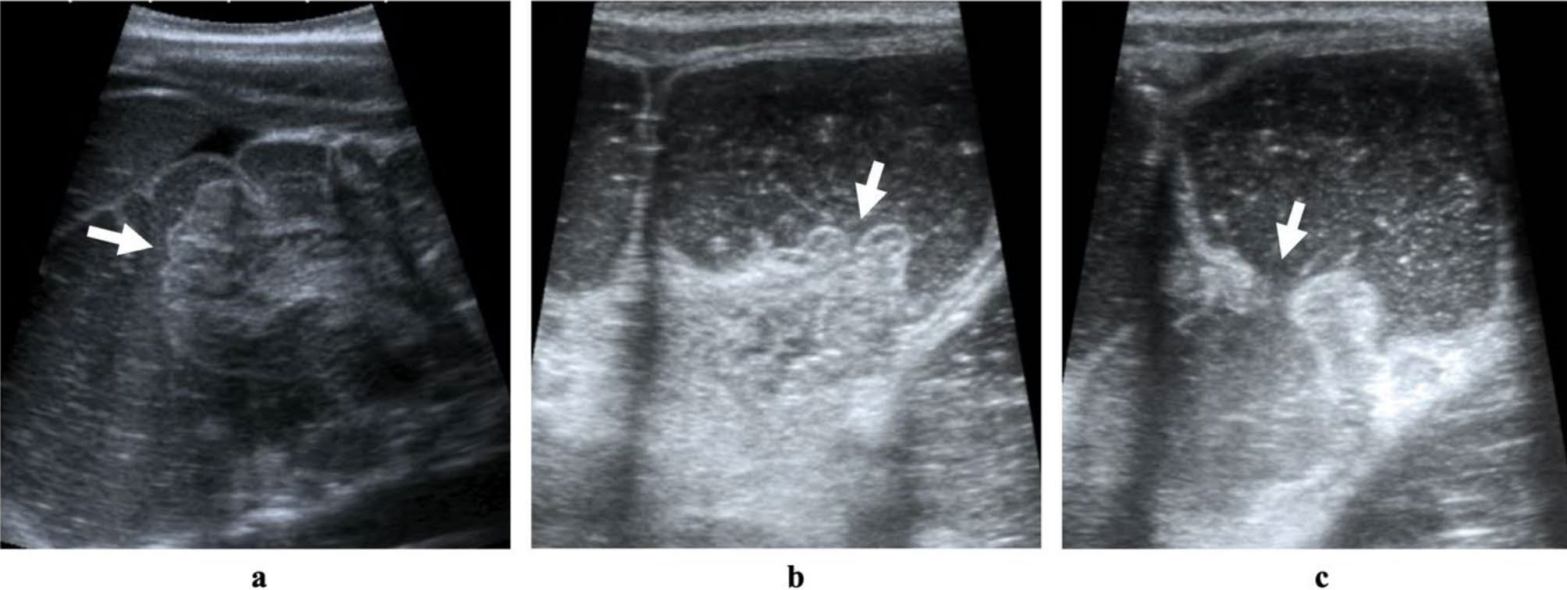
Grayscale static ultrasound images of US-guided hydrostatic reduction procedure. **a** Invaginated ileum (intussusceptum) marked with white arrow within the saline-filled colon (intussuscipiens) during the reduction procedure. **b** and **c** A successful resolution of the intussusception. **b** No invaginated ileum is seen within the colon, and the ileocecal valve (white arrow) is clearly visible. **c** The open ileocecal valve (white arrow) with reflux of saline into the ileum—a direct sign of successful reduction of the intussusception

During the procedure, the surgeon is pressing the child’s buttocks tight together to allow better sealing. By this method, a hydrostatic pressure of up to 120 mmHg within the lumen of the large bowel can be achieved. By filling the colon with saline, the pressure of the accumulated saline pushes the small bowel loop backward, forcing it to slowly exit the colon through the ileocecal valve. The whole process of intussusception reduction is continuously monitored by the radiologist with the US (Fig. 2). The inflow of saline through the ileocecal valve into the terminal ileum marks a successful reduction procedure. The reduction procedure is considered unsuccessful if all of the ileum cannot be hydrostatically pushed back through the ileocecal valve. In cases of unsuccessful US-guided hydrostatic reduction, when a partial reduction has been achieved and the clinical status of the child remains stable, another (delayed) attempt is made within 2–4 h after the previous attempt (up to 3 times, as long as the patient remains clinically stable).

### Statistical analysis

Data are presented as means, percentages, ranges, and interquartile ranges (IQR) when appropriate. Statistical analysis was conducted by GraphPad Prism 8 (GraphPad Software, San Diego CA, USA) [9]. The chi-square test was used for enumerable data comparison between the groups, while numeric data between the groups were compared by one-way analysis of variance (ANOVA). Differences were deemed statistically significant at *p* < 0.05.

## Results

We analyzed a total of 101 children who underwent the procedure of hydrostatic intussusception reduction under US guidance in our tertiary care hospital during this study period (11 years). There was a preponderance of males (71.3%), with a male-to-female ratio of 2.5:1. The age of children at the time of the procedure ranged from 2 to 126 months, with a mean age of 24.7 months (Table 1).

**Table 1:** Demographics data of patients who underwent the procedure of US-guided hydrostatic ileocolic reduction

		Group 1 (*n* = 49)	Group 2 (*n* = 25)	Group 3 (*n* = 27)	Total (*n* = 101)	*p* value
Gender: *n* (%)	Male	32 (65.3)	18 (72.0)	22 (81.5)	72 (71.3)	0.33^a^
	Female	17 (34.7)	7 (28.0)	5 (18.5)	29 (28.7)	
Age: mean, range (IQR) (months)		24.6, 3–92 (8–38)	23, 2–64 (6–41)	26.5, 3–126 (10–35.5)	24.7, 2–126 (8–38)	0.87^b^
Context: *n* (%)	Idiopathic	45 (91.8)	24 (96.0)	26 (96.3)	95 (94.1)	0.66^a^
	Secondary	4 (8.2)	1 (4.0)	1 (3.7)	6 (5.9)	

Group 1—reduction procedure was performed by pediatric radiologists; group 2—reduction procedure was performed by non-pediatric radiologists; group 3—reduction procedure was performed by radiology residents. The data are not statistically significant between the groups (^a^chi-square or ^b^one-way analysis of variance)

*IQR* interquartile range

There were 101 first attempts of US-guided hydrostatic reduction of ileocolic intussusception; among them, 49 attempts were performed by pediatric radiologists and 52 by non-pediatric radiologists or radiology residents. The group of subspecialized pediatric radiologists comprised 9 radiologists (range of working experience in pediatric radiology, 2–35 years) with the mean number of interventions per radiologist being 5.4 (range, 2–13) when accounting for the period of this study. The group of non-pediatric radiologists consisted of 13 radiologists, and the third group included 20 radiology residents. The mean number of interventions in the group of non-pediatric radiologists was 1.9 (range, 1–6) and the mean number of interventions for the group of radiology residents was 1.3 (range, 1–5). For 24 (72.7%) of these operators, this was their only time independently performing the procedure. Altogether, complete reductions were achieved in 76 first attempts with an overall success rate of 75.2%. The group of pediatric radiologists had 37 successful first attempts (75.5%), the group of non-pediatric radiologists had 19 (76.0%), and 20 (74.1%) successful first attempts, respectively. When comparing the first-attempt success rates of these groups, no statistically significant difference was found (*p* = 0.98). Of note, no event of the perforation occurred in either group during the reduction attempts (Table 2).

**Table 2:** Comparison of ileocolic reduction procedure parameters between pediatric radiologists, non-pediatric radiologists, and radiology residents

	Group 1 (*n* = 49)	Group 2 (*n* = 25)	Group 3 (*n* = 27)	Total (*n* = 101)	*p* value
First-attempt success rate: *n* (%)	37 (75.5)	19 (76.0)	20 (74.1)	76 (75.2)	0.98^a^
Second-attempt success rate: *n* (%)	1/9 (11.1)	1/2 (50.0)	*NP*	2/11 (18.2)	^b^
Recurrence rate: *n* (%)	10 (26.3)	4 (20.0)	1 (5.0)	15 (19.2)	0.17^a^
Presence of pathologic lead point: *n* (%)	4 (8.2)^c^	1 (4.0)^d^	1 (3.7)^e^	6 (5.9)	^b^

Group 1—reduction procedure was performed by pediatric radiologists; group 2—reduction procedure was performed by non-pediatric radiologists; group 3—reduction procedure was performed by radiology residents

*NP* not performed

^a^Data is not statistically significant between the groups (chi-square)

^b^Not compared between the groups

^c^All four patients had Meckel’s diverticulum

^d^Patient with Meckel’s diverticulum

^e^Patient with mesenteric cyst

Twenty-five first attempts of ileocolic intussusception reduction were unsuccessful (24.8%). In 11 of these 25 cases of failed first attempts at reduction, a delayed repeated reduction procedure was attempted. Most of the repeated attempts were performed by pediatric radiologists; non-pediatric radiologists and radiology residents rarely employed the repeated attempt. The repeated attempt was successful in two out of eleven patients. The second repeat attempt (i.e., the third attempt) was performed by two pediatric radiologists in only two children and was unsuccessful in both cases. In 3 of these patients with recurrent intussusception (4.2%), the intussusception recurred again after two or three successful reduction attempts and surgery was performed as the final treatment. The recurrence rate for pediatric radiologists, non-pediatric radiologists, and radiology residents was 26.3%, 20.0%, and 5.0%, respectively. Taken together, 27 patients (26.7%) underwent surgical treatment after failed reduction and/or early repeated recurrence, with their mean age of 17.3 months. Six of the 27 patients who required surgery had pathological lead points (5.9%); Meckel’s diverticulum was found in five patients and a mesenteric cyst in one child. The mean age of patients with a pathological lead point was 30.7 months. There were eight patients (16.3%) from the subspecialized group, seven patients (28.0%) from the non-subspecialized group, and six patients (22.2%) from the radiology residents group who had no lead point but necessitated surgery. The difference between the groups was statistically non-significant (*p* = 0.49). Necrotic bowel resection was required in three children (3.0%).

## Discussion

In this study, we compared the success rate of US-guided hydrostatic intussusception reduction procedure when performed by subspecialized pediatric radiologists, non-pediatric radiologists, and radiology residents. In all three groups, the reduction procedure proved to be effective and safe, with an overall success rate of 75.2% and no events of perforation. The pediatric radiologists, non-pediatric radiologists, and radiology residents had similar rates of successful reduction, specifically 75.5% vs. 76% vs. 74.1% successful first attempts, respectively. There was no statistically significant difference in success rates between the operator groups.

The results of our study indicate that the US-guided hydrostatic intussusception reduction procedure is a reliable and safe reduction technique, even in the hands of less experienced but trained operators such as non-pediatric radiologists and radiology residents. The mean number of procedures performed by an individual operator in the groups of non-pediatric radiologists and radiology residents was 1.9 and 1.3, respectively. For the majority of the non-pediatric radiologists and radiology residents (72.7%), that reduction attempt was the first time performing the procedure individually. It is an important finding that similar rates of success and safety were found among all operators during the US-guided hydrostatic intussusception reduction procedure because children with intussusception commonly present outside of regular work hours. It is difficult for hospitals to ensure the presence of an experienced pediatric radiologist to perform the procedure at all hours of every day, and children with intussusception are often transported to larger pediatric referral centers for an experienced (pediatric radiologist) operator to perform the intussusception reduction procedure. Consequently, the reduction procedure is often delayed (by the transport time), which presents a risk for the patient. The prolonged duration time of persistent, untreated ileocolic intussusception is associated with lower success rates of radiologically guided reduction and increased risk of ischemia, bowel necrosis, and/or perforation [10].

That said, it is important to stress that all three groups of operators in our study are highly skilled and trained in the use of US and educated on the US-guided hydrostatic reduction technique and recognition of radiological emergencies. Even the least experienced group of radiology residents had considerable training in this field, including six months of training at the abdominal radiology department, five months of sonography training, two months of pediatric radiology training, and at least a month of emergency radiology training. The operator’s ability to reliably recognize ileocolic intussusception by the US and to dynamically evaluate the movement of invaginated small bowel loops within the colon is vital for the success of this US-guided reduction procedure; therefore, the operator must be appropriately trained as mandated in our hospital. In addition, the ability to recognize US signs of complications, including bowel perforation, is also imperative.

At most hospitals, ileocolic intussusception reduction is most often performed by an experienced pediatric radiologist. If performed by a resident, most hospitals typically require supervision by an experienced pediatric radiologist [11]. Nevertheless, in several hospitals, radiology residents perform hydrostatic enema under US guidance independently, whereas experienced operators are often indispensable with fluoroscopy-guided air enema reductions [3, 11–14]. The results of our study demonstrate that trained radiology residents and non-pediatric radiologists are able to perform US-guided hydrostatic reduction without supervision and may facilitate the introduction of hydrostatic reduction procedure to centers that have a less experienced operator readily available and hence accelerate the process of intussusception reduction or make it available in the first place. Due to its greater affordability and availability, this technique may be even more valuable in developing countries, where radiologically guided reduction methods are not routinely used, and surgery is still the mainstay of the treatment in up to 88% of patients with ileocolic intussusception [15]. Fluoroscopy equipment is expensive, but ultrasound machines have in recent years become more accessible. In addition, US-guided intussusception reduction avoids exposure to potentially harmful ionizing radiation in this vulnerable pediatric population.

The success rate of ileocolic reduction in our study (75.2%) provides concordant results to other researchers using US-guided enema reduction of intussusception (57–89%) [4, 7, 16–20]. Since our center is the referral center in our country, a few complicated cases referred from other hospitals slightly decreased the overall success rate. Only a scarcity of studies previously researched the influence of radiologists’ experience on the success rate of ileocolic intussusception reduction. A study similar to ours was conducted by Crystal et al. and concluded that the experience of the operator who performs the procedure with water enema under US guidance significantly affects the outcome of the reduction. However, it is noteworthy that the number of operators and the number of attempted reductions included in their study was markedly lesser than in our study—their subspecialized pediatric radiologist team was comprised of only 3 members, and moreover, their non-subspecialized team performed overall only 13 attempts of ileocolic reduction [7]. The other published research evaluated the effect of operator experience on fluoroscopy-guided reductions. Shekherdimian and Lee reported that children who were presented to general (non-pediatric) hospitals who undergo fluoroscopy-guided contrast enema reduction have lower success rates compared to children’s hospitals [21]. Hence, the study indicates there could be an important difference between experienced and less experienced radiologists when performing fluoroscopy-guided reduction technique. Supporting the previous study, Bratton et al. reported that children treated in hospitals in the USA with smaller pediatric caseloads have a significantly greater risk of operative management compared to large pediatric hospitals [22]. This difference is likely related to more radiologic experience at large pediatric centers allowing for more proficiency with pneumatic reduction technique [22–24]. Contradictory, Tang et al. showed there is no significant difference in outcome if the fluoroscopy-guided pneumatic reduction is performed by an experienced radiologist or resident [8]. All in all, studies provide conflicting results from which it is difficult to draw an unambiguous conclusion about the effect of radiologists’ experience on performing the reduction procedure.

We acknowledge that this study has two main limitations. The first main limitation is a relatively small patient population. However, we would like to emphasize that our study has the largest patient population for evaluating the success rate of US-guided hydrostatic intussusception reduction between three independent groups; thus, we were able to obtain statistically validated results. Another limitation is that the patients were treated in a single center, which might be influenced by the local demographics or practice preferences of the institution. Nonetheless, we believe that we were able to achieve the consistent way of reducing intussusception by US among three groups of operators since our study was performed at a single center. The findings of the study promise a great potential to improve patient care by increasing access and decreasing time to the radiologic reduction of intussusception by the implementation of this method (US-guided hydrostatic reduction) in centers where radiologically guided intussusception reduction procedures are not readily available, particularly in general hospitals, hospitals without subspecialized pediatric radiologists, and in developing countries. Future multicenter studies with larger sample sizes will be helpful for confirmation of our findings.

## Conclusions

In conclusion, the results of our study suggest that US-guided hydrostatic reduction of ileocolic intussusception, as performed at our center, is a reliable, effective, and safe technique that achieves similar success rates when performed by a subspecialized pediatric radiologist, non-pediatric radiologist, or radiology resident in an academic setting, and after introductory education to the method.

## Data Availability

The authors will make the data supporting this article's conclusions available on request.

## Abbreviation

US: Ultrasound
